# Challenges of DHS and MIS to capture the entire pattern of malaria parasite risk and intervention effects in countries with different ecological zones: the case of Cameroon

**DOI:** 10.1186/s12936-018-2284-7

**Published:** 2018-04-06

**Authors:** Salomon G. Massoda Tonye, Celestin Kouambeng, Romain Wounang, Penelope Vounatsou

**Affiliations:** 1 Swiss Tropical and Public Health Institute, Basel, Switzerland; 2 University of Basel, Basel, Switzerland; 3 National Malaria Control Programme, Yaoundé, Cameroon; 4 National Institute of Statistics, Yaoundé, Cameroon

**Keywords:** Malaria, Malaria indicator survey, Demographic and health survey, Parasitaemia, Spatial correlation, Malaria interventions, Insecticide-treated nets, Rapid diagnostic test, Statistically important

## Abstract

**Background:**

In 2011, the demographic and health survey (DHS) in Cameroon was combined with the multiple indicator cluster survey. Malaria parasitological data were collected, but the survey period did not overlap with the high malaria transmission season. A malaria indicator survey (MIS) was also conducted during the same year, within the malaria peak transmission season. This study compares estimates of the geographical distribution of malaria parasite risk and of the effects of interventions obtained from the DHS and MIS survey data.

**Methods:**

Bayesian geostatistical models were applied on DHS and MIS data to obtain georeferenced estimates of the malaria parasite prevalence and to assess the effects of interventions. Climatic predictors were retrieved from satellite sources. Geostatistical variable selection was used to identify the most important climatic predictors and indicators of malaria interventions.

**Results:**

The overall observed malaria parasite risk among children was 33 and 30% in the DHS and MIS data, respectively. Both datasets identified the Normalized Difference Vegetation Index and the altitude as important predictors of the geographical distribution of the disease. However, MIS selected additional climatic factors as important disease predictors. The magnitude of the estimated malaria parasite risk at national level was similar in both surveys. Nevertheless, DHS estimates lower risk in the North and Coastal areas. MIS did not find any important intervention effects, although DHS revealed that the proportion of population with an insecticide-treated nets access in their household was statistically important. An important negative relationship between malaria parasitaemia and socioeconomic factors, such as the level of mother’s education, place of residence and the household welfare were captured by both surveys.

**Conclusion:**

Timing of the malaria survey influences estimates of the geographical distribution of disease risk, especially in settings with seasonal transmission. In countries with different ecological zones and thus different seasonal patterns, a single survey may not be able to identify all high risk areas. A continuous MIS or a combination of MIS, health information system data and data from sentinel sites may be able to capture the disease risk distribution in space across different seasons.

**Electronic supplementary material:**

The online version of this article (10.1186/s12936-018-2284-7) contains supplementary material, which is available to authorized users.

## Background

Malaria is an endemic disease and a public health issue in Cameroon. It is a major cause of morbidity and mortality among children less than 5 years. In 2014, the morbidity of malaria was 30% in children and 18% in adults [1, 2]. Conscious of this situation, the government has considered the fight against malaria to be a national priority and part of the health strategic plan [3]. Since 2002, the National Malaria Control Programme (NMCP) was created under the coordination of the ministry of public health. The aim was to improve the quality of strategic actions and to raise resources. During the last 10 years, huge investments have been deployed by donors, the international community and the government, to develop strategies and tools for reducing the burden of malaria in the country. According to the national malaria strategic plan of 2014–2018 [4], the NMCP is implementing interventions to sustain and scale up malaria control. Those interventions include distribution of insecticide-treated nets (ITN) to populations at risk and of sulfadoxine–pyrimethamine to pregnant woman, parasitological confirmation of suspected malaria cases (microscopy or rapid diagnostic test), and treatment of uncomplicated malaria cases by artemisinin-based combination therapy (ACT). Until 2011, the NMCP has distributed ITNs only to vulnerable groups. In 2012, the distribution policy has changed and more than eight million of long-lasting insecticide nets (LLIN) was given to populations at risk [5, 6]. Before the LLIN mass campaign distribution, two representative surveys were carried out by the National Institute of Statistics: a demographic and health survey (DHS) combined with multiple indicator cluster survey (MICS) and a malaria indicator survey (MIS).

The DHS was the first national malaria survey to collect prevalence data across the country, however for logistic reasons data were collected outside the malaria high transmission season. The NMCP and partners have decided to conduct the MIS during the second and most important rainy season (September–October), when the highest peak of malaria transmission occurs in order to assess the ability of DHS to estimate the malaria burden in the country [7]. Hence, the objective of this study is to assess the influence of the survey period on the detection of risk pattern by comparing estimates of the malaria parasite risk and the effects of interventions obtained from both surveys. The analysis was carried out using Bayesian geostatistical logistic regression models similar to the ones that have been used for spatial analyses of other DHS and MIS data such as Angola, Senegal, Nigeria, Burkina Faso, Uganda and Sudan [8–13].

## Methods

### Country profile

Cameroon is a central Africa country, bordered with Nigeria to the West, Chad to the North, Central African Republic to the East, Congo, Gabon and Equatorial Guinea to the South. The country is decentralized and organized around 10 regions, 58 divisions and 360 communal areas. English and French are the official languages. Yaoundé is the political capital and Douala is the economic town. The global surface of the country is 475,650 km^2^, population is around 22 million inhabitants [14, 15] and index of human development is 0.512 in 2015 [16]. The percentage of the population living in urban areas is 49%. Children under 5 years old represent 17% of the population [3, 17]. Despite of the presence of natural resources as oil, gas, iron, gold, and favourable climatic situations for agriculture, the national income per inhabitant is still low (< 2000$ per year) with important disparity between urban and rural areas [18]. The country has different geographic and ecological zones, that generate six epidemiologic facets of malaria transmission [19–21], corresponding to different ecological systems: the dry Sahelian in the Far North region and the Sudano-guinean in the North region where malaria transmission period is between 4–6 months; the highlands of Adamawa and West regions with length of malaria transmission between 7–12 months; the equatorial forests which includes Centre, East and part of South regions where the transmission is stable; and the Atlantic coastal covering the Littoral and a part of South and South-West regions where the malaria is perennial with seasonal variations. The malaria transmission in the North part of Cameroon is characterized by seasonal pattern linked to rainy season which cover the period from August to October. Like many Africa countries, *Plasmodium falciparum* is also the predominant species and responsible of more than 95% of confirmed infection cases in this study [22, 23].

### Malaria parasitological data

#### DHS-MICS 2011 survey

**Fig. 1:**
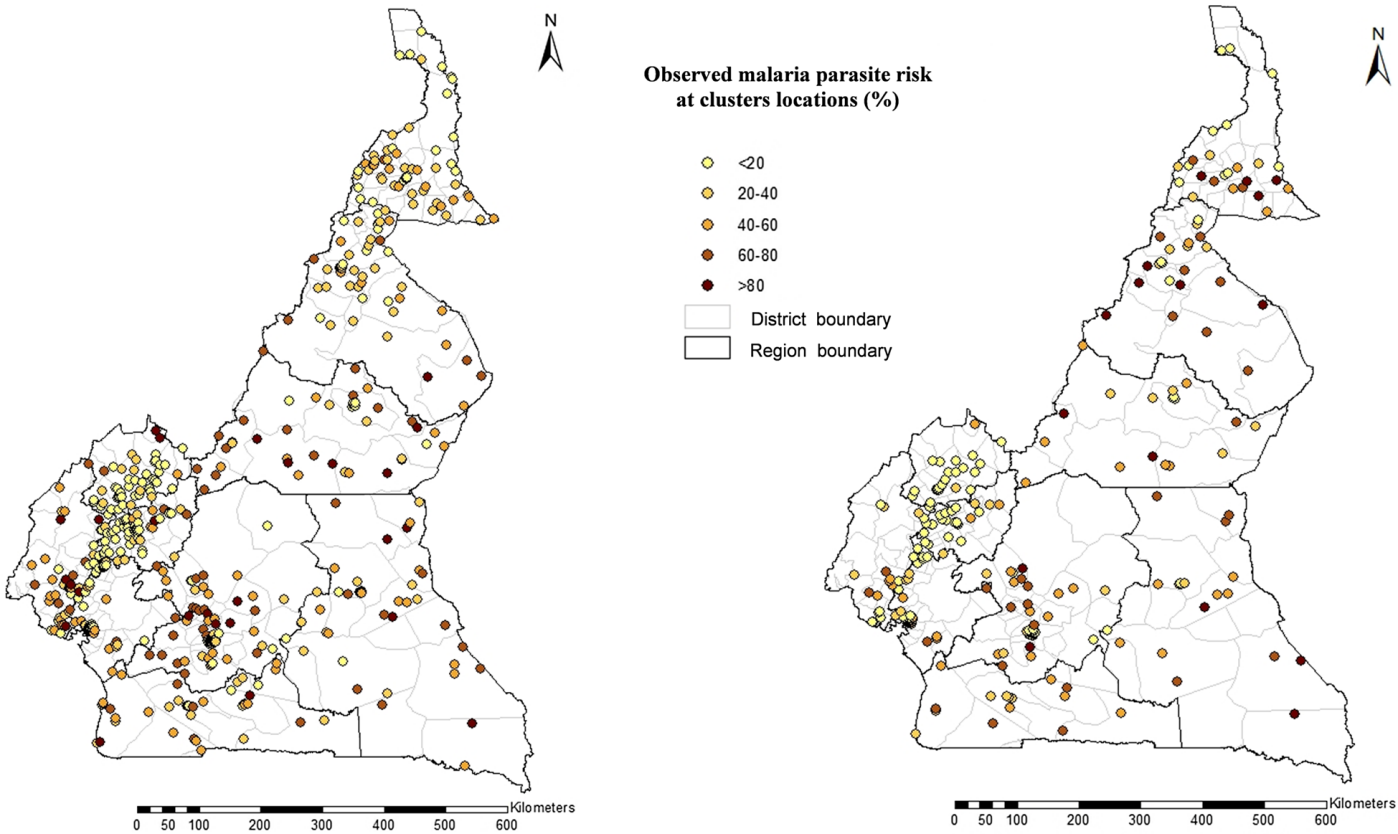
Observed malaria parasite risk in children under 5 years at 580 DHS locations (left) and at 257 MIS locations (right)

DHS are nationally representative household based surveys commonly carried out by the National Institute of Statistics and ICF International in Africa or elsewhere collecting socioeconomic, demographic, disease and intervention related data. MICS is another standardized household survey carried out by UNICEF, compiling health related data on children and women. Both DHS and MICS were carried jointly in Cameroon during January–August 2011. A sample of 15,050 households living in 580 clusters was selected using a two-stage sampling approach, 291 clusters were in urban zone (Fig. 1). Blood samples were taken in 50% of households inside the cluster surveyed and 5515 children that are under 5 year were tested by a rapid diagnostic test (SD BIOLINE Malaria Antigen Pf/Pan) [3].

#### MIS 2011 survey

The MIS was carried out between September and November 2011, during the malaria high transmission season, 1 month after the increase of rains in the country. The MIS was conducted on 6040 households within 257 clusters randomly selected out of the 580 clusters of the DHS 2011 (Fig. 1). The sample size was determined using the same calculations as DHS, however it was based on the proportion of children aged 0–59 months using ITN in comparison to DHS that considered the proportions of a range of indicators. Malaria screening was performed in 4939 children under 5 years old living in selected households and with the approval of adult in charge using a rapid diagnostic test (First Malaria Response Antigen) [24].

### Environmental and climate factors

Environmental and climate predictors were extracted from satellite sources (Table A.1 in Additional file 1). In particular, data were compiled on land surface temperature during the day and night (LSTD, LSTN), Normalized Difference Vegetation Index (NDVI), Enhanced Vegetation Index (EVI), land cover surface and permanent water bodies obtained from the moderate resolution imaging spectroradiometer (MODIS) terra satellite. rainfall estimates (RFE) and altitude were retrieved from FEWS (or Famine Early Warning Systems Network) and SRTM (or Shuttle Radar Topographic Mission) web sites, respectively [25, 26]. Climatic proxies with weekly and bi-weekly temporal resolution were averages over the 1 year period prior to the survey.

### Socio-economic factors

Socio-economic data were included in both, the DHS and the MIS surveys. Two socio-economic proxies were used, education of women in reproductive age and household asset index. The education level was treated as a categorical variable with three levels (primary, secondary and university). The household asset index was included in the database and used in categorical form, grouped into quintiles corresponding to the poorest, poor, middle, rich and richest segments of the population. Rural and urban area information was available in the database for the observed survey locations and it was extracted from the GRUMP (or Global Rural and Urban Mapping Project) database at the locations of predictions [27].

### Interventions

To capture the effects of interventions at national level, output indicators were generated using data available in the DHS and MIS, according to the household survey indicators tool for malaria control developed by Roll Back Malaria and partners. In particular, the following coverage indicators of use and access to ITN interventions were created: (a) proportion of children under 5 years old who slept under an ITN the previous night; (b) proportion of households in the cluster with at least one ITN; (c) proportion of households in the cluster with at least one ITN for every two people; (d) proportion of population with access to an ITN within their household. Furthermore, a health system performance indicator was calculated to measure the proportion of children under 5 years old with fever in the last 2 weeks who seek treatment at hospital, tested and treated with recommended ACT [28].

### Bayesian geostatistical modelling

Bayesian geostatistical binomial models fitted on cluster level malaria aggregated data were used to estimate parasitaemia risk at high spatial resolution based on climatic predictors (Model 1). Climatic variables were categorized in groups with cut-offs defined from quintiles and exploratory analysis. Geostatistical variable selection was carried out to identify the most important climatic and environmental predictors, including their best fitting functional form [29, 30]. For each predictor a categorical indicator was introduced with values 0, 1 and 2 corresponding to exclusion of the predictor from the model or inclusion in linear or categorical form, respectively. It was assumed that the indicator arose from a multinomial distribution with probabilities defining the variable-specific exclusion/inclusion probabilities (in linear/categorical forms) in the model (Additional file 2). A threshold of 50% was considered for the probability of inclusion (i.e. posterior inclusion probability) into the predictive geostatistical model. In the final model, the effect of a predictor was considered to be statistically important if the 95% Bayesian credible interval (BCI) of the coefficient did not include the one on the odds ratio scale. Validation of Model 1 was performed to assess the model’s predictive performance. In particular, the sample was divided into a training set which included 80% of the data which was used for model fit and a test set consisting of the remaining data. Model validation compared the mean error between the observed parasitaemia at the locations of the test set with the model-based predicted risk. The model predictive performance was also evaluated by calculating the proportion of test locations correctly predicted within the 95% of BCI. Bayesian kriging was applied using Model 1 to predict the parasitaemia risk over a gridded surface of 117,192 cells and obtain pixel-level risk estimates at 2 × 2 km^2^ resolution [31, 32].

Geostatistical variable selection was also applied to select the most important coverage indicators of malaria interventions. A Bayesian geostatistical Bernoulli model was fitted on the parasitaemia status of each child to estimate the effect of selected malaria interventions (Model 2) after adjusting for potential confounding effects of the climatic factors used in Model 1 and of the socioeconomic factors. The same methodology was employed separately on the DHS and the MIS data. Model fit and prediction were conducted in R [33] and OpenBUGS version 3.2.3 (Imperial College and Medical Research Council, London, UK) [34, 35]. Convergence of parameters was assessed by the Geweke statistic and by visually inspecting the traceplots [36]. Computations were performed in the parallel scientific computing (sciCORE) platform of Basel University. Different maps were produced by ESRI’s ArcGIS version 10.2.1 for Desktop (http://www.esri.com/).

## Results

### DHS results

The observed parasitaemia risk in children under 5 years old was 30% at national level, 37% in the rural and 20% in the urban areas. In urbanized cities, such as Yaoundé and Douala the malaria parasite risk was among the lowest in the country, i.e. 12 and 13%, respectively. Five percent of the population had access to an ITN within their household and 21% of children slept under an ITN during the night preceding the survey. The percentage of children under 5 years old with fever in the last 2 weeks that treated with ACT was 6%. The proportion of children from the poorest and poor quintiles was 63% and the proportion of mothers with at least primary education was 80% (Table 1).

**Table 1:** Descriptive information of the DHS and MIS data

Survey information	DHS	MIS
Rainy period	Low rainy season: April, May, June High rainy season: August, September, October	
Survey period	January to August	September to November
Numbers of locations	580	257
Numbers of households	15050	6040
Numbers of children aged 0–59 months surveyed	5515	4939
Parasitaemia prevalence	30 (12–53)	33 (6–57)
Socio economic		
Education level of mothers (%)		
No education	20	23.2
Primary	33.8	30.2
Secondary	40.7	39.9
University	5.5	6.7
Wealth index (%)		
Most poor	22	25
Very poor	21.8	22.2
Poor	20.2	21.2
Less poor	19.7	17.5
Least poor	16.3	14
ITN ownership		
Percentage of households with at least one ITN	36.4 (26.5–52.3)	46.3 (30–61.9)
Percentage of households with at least one ITN for every two person	14 (8.6–27.6)	15 (6.8–26.9)
ITN use, ACT and indoor residual spray coverage		
Percentage of children aged 0–59 months who slept under an ITN the night before the survey	21 (5.4–38.6)	35 (9.7–56.2)
Percentage of population with access to an ITN in their household	5 (3–9.7)	9 (2.7–13. 8)
Indoor residual spray	2.3 (0–8.9)	1.9 (0.2–5.2)
Percentage of children with fever in the last 2 weeks who seeked treatment and received ACT	6.1 (0.7–17.8)	12.6 (1.1–29.8)

The geostatistical variable selection identified NDVI and altitude (in categorical form) as the most important predictors of parasitaemia risk, using the cluster level model (Model 1). The proportion of the population with access to an ITN in their household and the proportion of children under 5 years old with fever in the last 2 weeks treatment-seeking at hospital, tested and treated with ACT, were selected from Model 2 (Table 2).

**Table 2:** Posterior inclusion probabilities (%) of the climatic predictors and intervention coverage indicators obtained by the geostatistical variable selection applied to DHS and MIS data

Model	Variable	DHS			MIS		
		Excluded	Continuous form	Categorical form	Excluded	Continuous form	Categorical form
Model 1, 2: cluster level	Rainfall	58	42	0	36	18	46
	NDVI^a^	14	*86*	0	12	*83*	5
	LSTD	80	20	0	55	23	22
	EVI^a^	56	32	12	16	17	*67*
	Distance to water body^a^	41	33	26	23	25	*52*
	Altitude^a^	0	0	*100*	1	*96*	3
	LSTN	43	10	47	54	29	17
	Forest^a^	69	–	31	34	–	*66*
	Savana	58	–	42	69	–	31
	Cropland	82	–	18	72	–	28
Model 2: individual level	% population with access to an ITN in their household^a^	17	*83*	–	42	*58*	–
	% households with at least one ITN	62	38	–	64	36	–
	% children slept under ITN previous night	61	39	–	36	*64*	–
	% of households with one ITN per two persons^a^	52	48	–	33	*67*	–
	% of children with fever who received recommended anti-malarial drugs (ACT)^a^	46	*54*	–	70	30	–

^a^Variable with posterior inclusion probability (continuous or categorical) above 50%

**Fig. 2:**
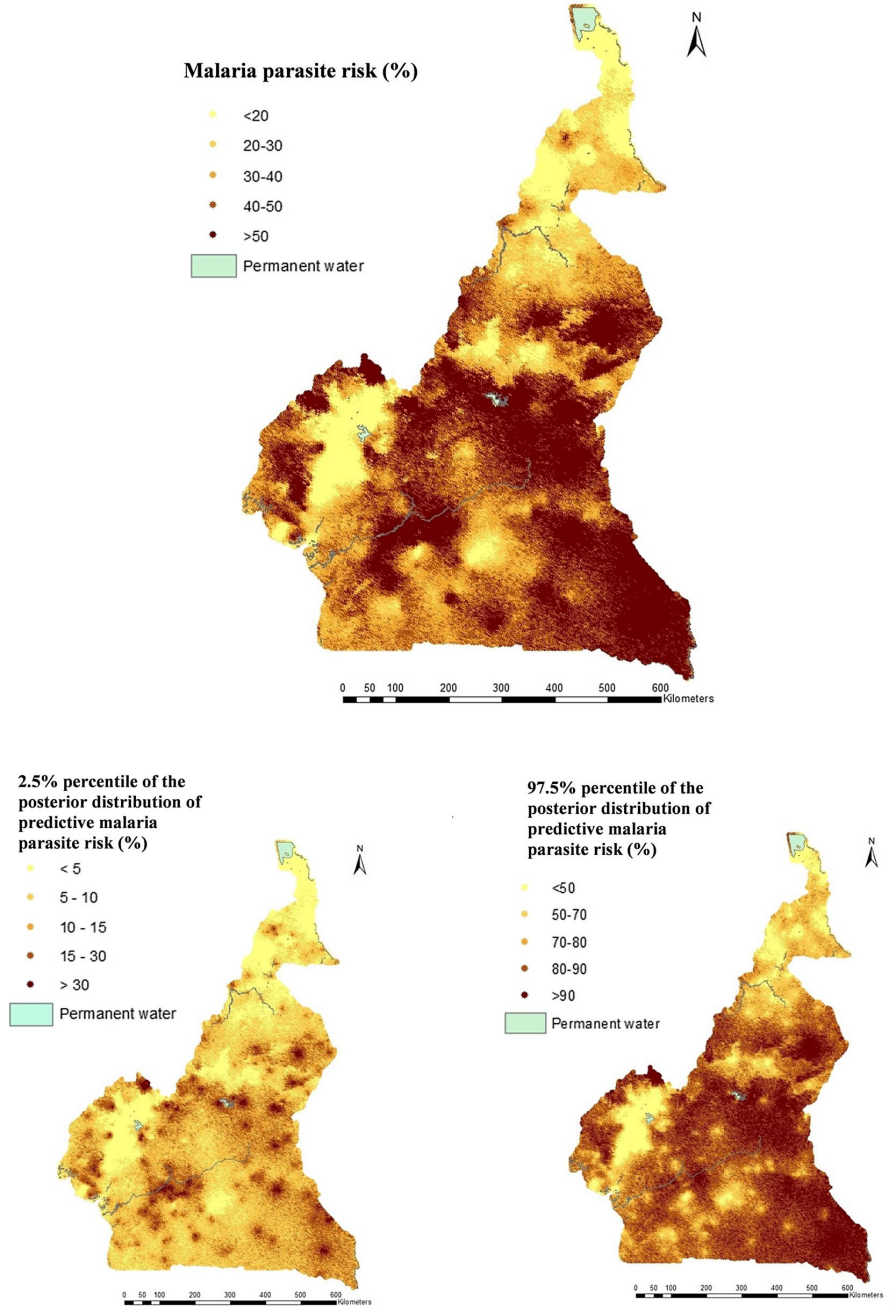
Malaria parasite risk estimates among children less than 5 years, obtained from Model 1 using the DHS 2011; median (top), 2.5th percentile (bottom left) and 97.5th percentile posterior predictive distribution (bottom right)

Posterior estimates of the model’s parameters are shown in Table 3. The climatic, cluster level model confirmed known relations between malaria parasite risk and climatic predictors, i.e. a positive association with NDVI and a negative relation with altitude. A malaria parasite risk map was generated using the climatic predictors identified from the DHS data (Fig. 2).

**Table 3:** Estimates (posterior median and 95% BCI) of the geostatistical model parameters based on the cluster level climatic (Model 1) and the individual level model (Model 2), DHS 2011

Factor	DHS	
	Model 1	Model 2
	OR (95% BCI)	OR (95% BCI)
NDVI	1.41 (1.19; 1.68)	1.22 (0.99; 1.5)
Altitude (m)		
< 1000	1	1
1000–1500	0.43 (0.26; 0.67)	0.37 (0.22; 0.61)
> 1500	0.26 (0.11; 0.60)	0.14 (0.05; 0.35)
Sex		
Female		1
Male		1.03 (0.90; 1.18)
Area type		
Rural		1
Urban		0.72 (0.53; 0.96)
Wealth index		
Most poor		1
Very poor		0.84 (0.66; 1.08)
Poor		0.91 (0.67; 1.23)
Less poor		0.78 (0.54; 1.12)
Least poor		0.32 (0.21; 0.49)
Education level of mothers		
No education		1
Primary		0.83 (0.69; 0.99)
Secondary		0.68 (0.55; 0.85)
University		0.47 (0.24; 0.89)
Age		
0–1^a^		1
1–2		1.83 (1.43; 2.37)
2–3		2.29 (1.78; 2.94)
3–4		2.92 (2.28; 3.77)
> 4		3.10 (2.41; 3.99)
% population access to an ITN in their household		0.23 (0.07; 0.74)
% of children with fever in the last 2 weeks who received ACT		1.33 (0.99; 1.76)

Spatial parameters	Posterior median (95% BCI)	Posterior media (95% BCI)
σ^2^	1.42 (1.09; 1.55)	1.43 (1.08; 1.97)
Range (km)	65.09 (44.90; 74.29)	81.63 (54.20; 134.54)

^a^Children less than 6 months were not surveyed

The individual level model (Model 2 in Table 3) shows that children in urban areas or those living in households with higher socioeconomic level were less affected by malaria. Children to mothers with high educational level or aged below 12 months had low malaria parasite risk. The proportion of population with access to an ITN in their household was able to capture a statistically important effect on parasitaemia risk.

### MIS results

The national observed malaria parasite risk was 33% with substantial disparities between rural (43%) and urban (19%) areas. The North-West region and the towns of Yaoundé and Douala had registered low prevalence of 10, 6 and 16%, respectively. According to the survey data, 9% of the population had access to an ITN within their household and 15% of households possessed one ITN per two persons. The percentage of children under 5 years old with fever in the last 2 weeks that received ACT was 12%. The percentage of children from the poorest and poor quintiles was 68% and the proportion of mothers with at least primary education was 76% (Table 1).

The geostatistical variable selection applied to the cluster level model (Model 1) had identified NDVI, the categorical forms of EVI and of distance to water, the presence of forest and altitude as the most important predictors of parasitaemia risk. The individual level model estimated high posterior inclusion probabilities for the following ITN coverage proxies, i.e. the proportion of population with access to an ITN in their household, the proportion of children who slept under an ITN in the previous night and the proportion of households with one ITN per two persons. However, the paired correlations between the above ITN indicators were ranging from 0.6 to 0.8; therefore, the indicator included in the final model (Model 2) was the last ITN coverage measure which had the highest inclusion probability (Table 2).

**Fig. 3:**
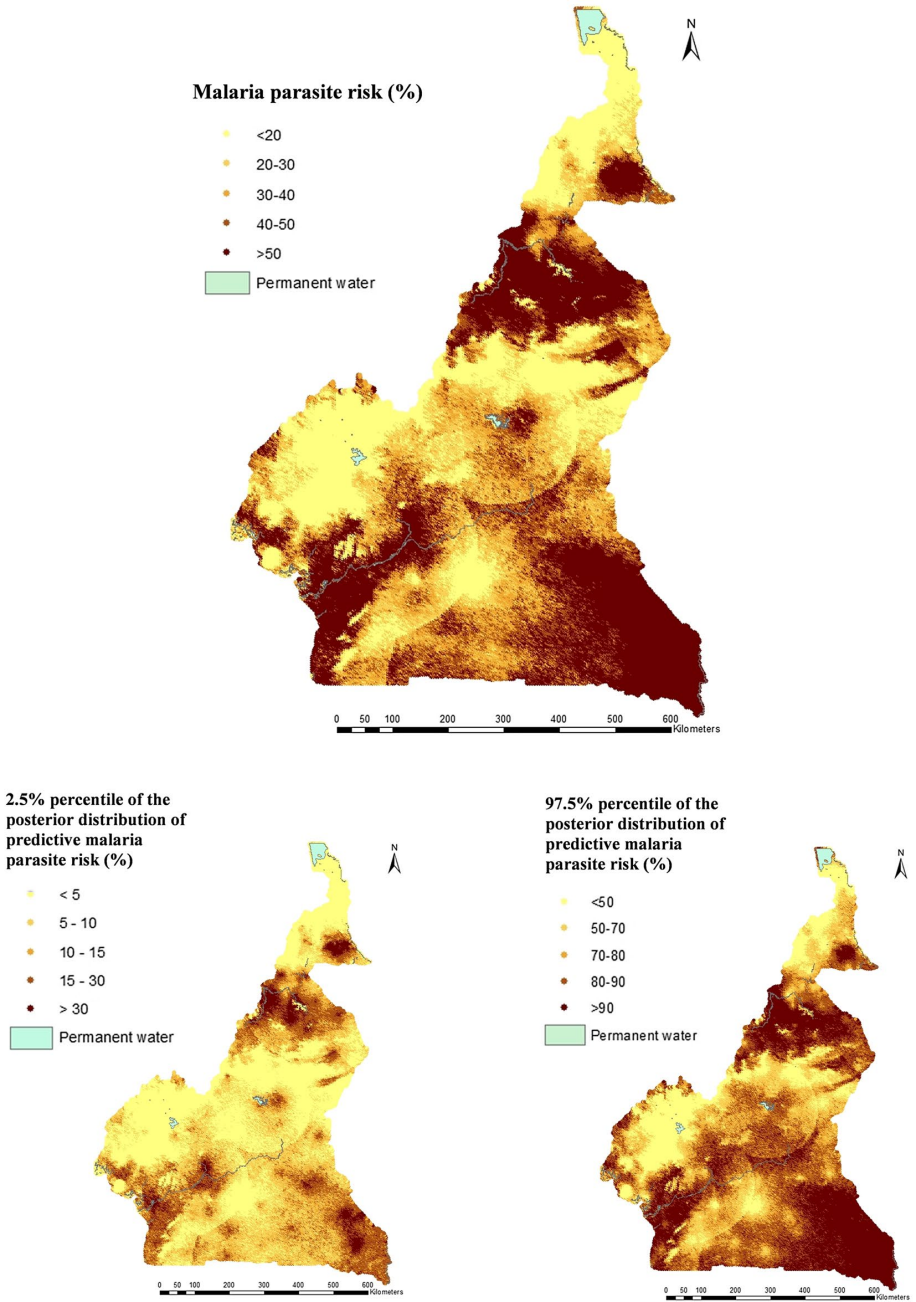
Malaria parasite risk estimates among children less than 5 years, obtained from Model 1 using the MIS 2011; median (top), 2.5th percentile (bottom left) and 97.5th percentile posterior predictive distribution (bottom right)

Parameter estimates for Model 1 and 2 are shown in Table 4. The cluster level predictive model indicated that malaria parasite risk was positively related with NDVI, EVI and presence of forest, and it was negatively associated with altitude. A malaria parasite risk map was drawn with climatic predictors selected by the MIS (Fig. 3).

**Table 4:** Estimates (posterior median and 95% BCI) of the geostatistical model parameters based on the cluster level climatic (Model 1) and the individual level model (Model 2), MIS 2011

Factor	MIS	
	Model 1	Model 2
	OR (95% BCI)	OR (95% BCI)
NDVI	1.55 (1.12; 2.12)	1.33 (0.97; 1.82)
EVI		
< 0.21	1	1
0.21–0.38	1.90 (1.03; 3.51)	1.38 (0.82; 2.33)
> 0.38	1.25 (0.51; 3.02)	0.92 (0.41; 2.1)
Distance to water body (m)		
< 70	1	1
≥ 70	1.82 (1.005; 3.45)	1.60 (0.90; 2.86)
Altitude	0.39 (0.26; 0.57)	0.37 (0.25; 0.53)
Forest		
No	1	1
Yes	1.55 (1.002; 2.39)	1.17 (0.77; 1.79)
Sex		
Female		1
Male		0.99 (0.86; 1.15)
Area type		
Rural		1
Urban		0.55 (0.38; 0.80)
Wealth index		
Most poor		1
Very poor		0.6 (0.46; 0.76)
Poor		0.66 (0.49; 0.89)
Less poor		0.46 (0.32; 0.66)
Least poor		0.39 (0.25; 0.61)
Education level of mothers		
No education		1
Primary		1.15 (0.92; 1.43)
Secondary		0.92 (0.70; 1.22)
University		1.03 (0.57; 1.84)
Age		
0–1^a^		1
1–2		1.31 (0.96; 1.77)
2–3		2.29 (1.70; 3.10)
3–4		2.57 (1.90; 3.48)
> 4		3.49 (2.62; 4.65)
% households with 1 ITN per 2 persons		0.16 (0.05; 0.47)

Spatial parameters	Posterior median (95% BCI)	Posterior median (95% BCI)
σ^2^	1.81 (1.24; 2.92)	1.62 (1.10; 2.76)
Range (km)	154.8 (89.50; 292.96)	188.09 (100.35; 353.63)

^a^Children less than 6 months were not surveyed

The individual level model (Model 2 in Table 4) showed that children in rural areas as well as those living in households with lower socioeconomic status are more vulnerable to parasitaemia risk. Children aged below 12 months have low risk. The educational level of mother was not statistically associated with malaria parasite risk. ITN coverage was statistically important and had a negative effect on malaria parasite risk.

**Fig. 4:**
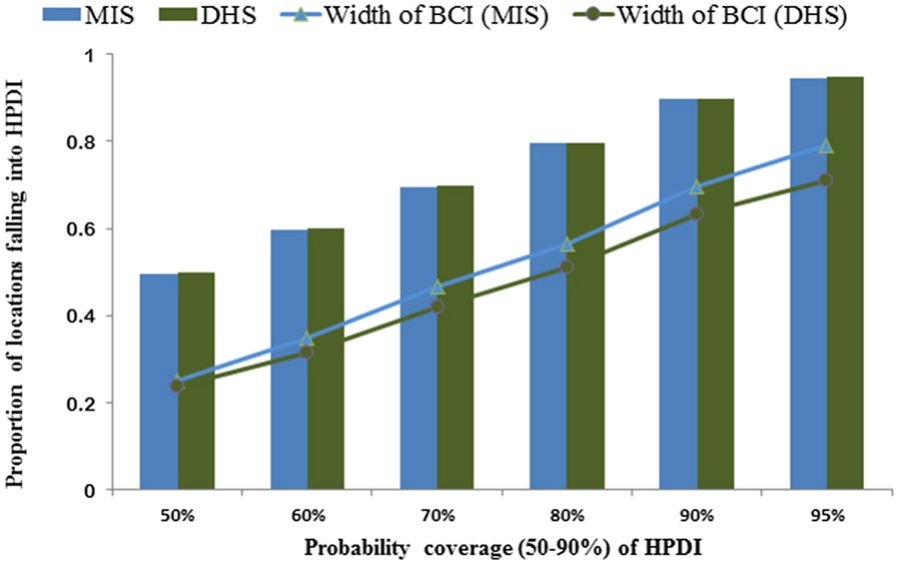
Proportion of test locations falling within highest posterior density intervals (HPDIs) of varying probability coverage

The proportion of test locations falling into the BCIs of the predictive posterior distributions with probability coverage varying from 50 to 95% was comparable for both surveys (Model 1), but the accuracy of estimates was higher for the DHS data as it is shown by the smallest BCI width (Fig. 4) which indicates the difference between the upper and the lower values of the interval. Validation of the cluster level Model 1 on the DHS and MIS data showed a mean absolute error of 0.050 and 0.038, respectively.

## Discussion

This study is the first to assess the influence of survey season on the estimates of the geographical distribution of malaria parasite risk and of the effects of interventions, using data collected by DHS and MIS carried out at the same locations and year, but at different malaria transmission seasons. The analysis employed Bayesian geostatistical models because this study was interested in comparing the estimates of the risk pattern across the country rather than at the observed locations.

The DHS collects a large number of indicators on diverse sectors and huge logistics are involved to guarantee the coverage of all clusters, in particular those in rural areas with difficult access. Moreover, the planning of DHS usually avoids the rainy season in Africa because of road’s degradation which challenges the survey implementation. The constraints described above have often an impact on the schedule and duration of DHS. The DHS and MIS surveys in Cameroon provide a unique opportunity to assess the effect of season on malaria survey-based estimates.

Both surveys showed low level of parasitaemia risk (under 5%) in West and Adamawa highlands. These areas are suitable for elimination interventions. Also, both data indicated that, the parasitaemia risk in East region was the highest in the country and above 50%. This high risk level is explained by the important coverage of forest, the predominance of rural areas and the low educational level of the population.

DHS data did not identify a cluster of high malaria parasite risk in the North and Far-North regions as estimated by the MIS. However, evidence from the upsurge of malaria cases that over strain the capacity of the health system during the rainy season and the high malaria mortality risk among children in the northern part of the country does not support the DHS finding [37, 38]. The non-concomitance between DHS and the malaria seasonal transmission in the north regions may explain the underestimation of malaria parasite risk in that area.

Furthermore, DHS could not capture a malaria cluster in the coastal part which is the estuary of the biggest rivers in the country that pour into the Atlantic Ocean. During the long rainy season that begins in August, some areas are flooded and large ponds of stagnant water are created [39–42]. The high transmission occurs just within the rainy season which is characterized by the increase of mosquito population. The water availability is among the key criteria for mosquito breeding, especially in the North part of Cameroon which is covered by the Sahel and in coastal towns, such as Douala because of poor condition of the pluvial drainage system. The model has identified additional climatic factors in MIS compared to the DHS.

In the Adamawa, North-West, West and Centre regions of the country, MIS estimated lower risk compared to DHS. In the capital, Yaoundé, parasitaemia risk was 6% based on MIS that is half the one obtained by the DHS data. The coverage of household by an ITN among the population of Yaoundé and Douala was 31 and 37%, respectively. Among households with at least one ITN, the percentage of those who use ITN in Yaoundé and Douala was among the highest in the country, i.e. 43 and 52%, respectively. Human behaviour at the beginning of the rainy season changes and people are likely to increase the use of preventive tools, such as ITN, mosquito spraying devices or repellents [43–45].

The altitude and NDVI were identified as important predictors in the cluster level models of both surveys. The presence of forest, EVI and distance to water body were found to be important in modelling the MIS data. As known, the altitude has a negative effect on malaria parasite risk. The effect of distance to water was not linear and households located more than 70 m away from water bodies are at higher risk of malaria compared to those households close to them for a number of reasons including the wind direction and the availability of human hosts [46]. Rainy season has an influence on vegetation and on human activities, such as farming which exposes people to mosquito bites, and that could be the reason of the positive association between the EVI, NDVI, the presence of forest and the parasitaemia risk [47, 48].

The analysis of the MIS data showed that the proportion of households with one ITN per two persons was statistically important with a negative effect indicating that the household coverage had an influence on malaria parasite risk among children [49]. According to the DHS, the ITN coverage indicator with a statistically important and protective effect was the population with access to an ITN. The use of ACT among children under 5 years old with fever in the last 2 weeks before the survey was positively associated to the malaria parasite risk but not statistically important. Similar results regarding ACT have been obtained from the MIS in Uganda and in Burkina-Faso [11, 12].

The disease risk resembles the pattern of socioeconomic inequalities in the country. In both surveys, the place of residence had an important effect and was negatively associated to malaria parasite risk. The DHS data showed that the effect of only the least poor category of the wealth index was statistically important compared to the most poor baseline category, however the MIS data estimated statistically important effects in all socio-economic categories. The educational level of mothers had a protective effect which was however statistically important only for the DHS. These results suggest that during the high malaria transmission season, the quality of the household environment is more important than the mother’s education. Obviously, children from wealthy households can benefit from additional vectors control tools, such as appropriate malaria treatment, ITNs, sprays products and the sanitized neighbourhood. Wanzirah et al. and Tusting et al. have also shown that high house quality reduces the entry of mosquito vectors and, therefore, lessens the risk of infection [50, 51].

A gradient of malaria parasite risk was associated to the age and as expected the gender effect was not statistically important. Younger children were at lower risk than older ones, which may be a consequence of the passive immunity given by mothers [52].

The high residual spatial correlation estimated by the models, especially those that used the MIS data indicates the presence of unmeasured spatially structured factors that influence the geographic distribution of the parasitaemia risk. It is likely that the climatic proxies considered in the model such as day and night LST or NDVI and EVI were not able to capture the entire ground climatic conditions. Similar analyses of other MIS data estimated relative high residual spatial correlation, particularly in recent surveys that climatic factors are confounded from malaria interventions [10, 12, 30]. The BCI width of the estimated parameters obtained with DHS were tighter than those of MIS, most likely due to the smaller number of survey clusters in the later [53, 54].

Both, DHS and MIS were used a RDT. RDTs could remain positive for few weeks after a malaria treatment. Therefore our estimates of parasitaemia risk may be slightly overestimated than those based on diagnosis by microscopy [55–57].

DHS and MIS are based on a two-stage cluster sampling design. In the first stage, the number of clusters that are selected at regional level is proportional to the population. This design oversamples clusters in places with high population density and can selects fewer clusters over larger regions with small populations (i.e. East region) where the disease may vary more compared to the urban areas and big cities such as Yaoundé and Douala. Therefore, the DHS/MIS survey design may provide lower precision of the estimates in rural areas.

Since 2011, Cameroon has implemented two mass campaigns of LLINs, introduced preventive treatment of children against malaria in the North region and built two large dams in the East and South regions. There is currently a DHS ongoing in Cameroon and the results of this study will serve as a baseline to assess the changes in malaria risk as a result of disease interventions, climatic effects and environmental modifications [58, 59].

## Conclusion

Timing of the malaria survey influences estimates of the geographical distribution of the disease risk, especially in settings with seasonal transmission. The DHS and MIS in Cameroon provide information about the geographical distribution of malaria parasite risk and of the effects of interventions in a country that different ecosystems cohabitate. In countries where malaria transmission is affected by seasonality, a single survey may not be able to identify all high-risk areas. A continuous MIS similar to the one running for example in Senegal or a combination of MIS, health information system data and data from sentinel sites may be able to capture the disease distribution in space across different seasons. However, in countries with no variation in the malaria transmission season, a single survey may be sufficient.

## Additional files

**Additional file 1.** Sources, spatial and temporal resolution of predictors.

**Additional file 2.** Geostatistical models formulation.

## Abbreviations

ACT: artemisinin-based combination therapy
BCI: Bayesian credible interval
DHS: demographic and health survey
EVI: Enhanced Vegetation Index
FEWS: Famine Early Warning Systems Network
GRUMP: Global Rural and Urban Mapping Project
HPDI: highest posterior density intervals
ITN: insecticide-treated net
LLIN: long-lasting insecticide net
LSTD: land surface temperature during the day
LSTN: land surface temperature during the night
MICS: multiple indicator cluster survey
MODIS: moderate resolution imaging spectroradiometer
NDVI: Normalized Difference Vegetation Index
NMCP: National Malaria Control Programme
RDT: rapid diagnostic test
RFE: rainfall estimates
SRTM: Shuttle Radar Topographic Mission
UNICEF: United Nations International Children’s Emergency Fund
