# Supplementary material for: Challenges of DHS and MIS to capture the entire pattern of malaria parasite risk and intervention effects in countries with different ecological zones: the case of Cameroon
Source: Malar J. 2018 Apr 6;17:156. doi: 10.1186/s12936-018-2284-7 (PMC5889563; doi:10.1186/s12936-018-2284-7)
Supplement: Supplementary file 1 — Additional file 1. Sources, spatial and temporal resolution of predictors. [file 12936_2018_2284_MOESM1_ESM.docx]

**Additional file 1**

Table A. 1 : Sources, spatial and temporal resolution of predictors

| **Data** | **Spatial resolution (km²)** | | **Period** | | **Temporal**  **resolution** |
| --- | --- | --- | --- | --- | --- |
| Land surface temperature (LST) for day and night ^1^ | 1 × 1 | 2010-2011 | | 8 days | |
| Normalized difference vegetation index (NDVI) ^1^ | 0.25 × 0.25 | 2010-2011 | | 8 days | |
| Enhanced vegetation index (EVI)^1^ | 1 × 1 | 2010-2011 | | 16 days | |
| Rainfall estimates(RFE) ^2^ | 8 × 8 | 2010-2011 | | 10 days | |
| Land cover ^1^ | 1 × 1 | 2010-2011 | | year | |
| Digital elevation (Altitude) ^3^ | 1 × 1 | 2011 | | NA | |
| Permanent water bodies (rivers, lakes, wetlands) ^1^ | 1 × 1 | 2011 | | NA | |

1<https://reverb.echo.nasa.gov/reverb>

2 <https://earlywarning.usgs.gov/fews/>

3 <http://glcfapp.glcf.umd.edu/data/srtm/>
