# Supplementary material for: Challenges of DHS and MIS to capture the entire pattern of malaria parasite risk and intervention effects in countries with different ecological zones: the case of Cameroon
Source: Malar J. 2018 Apr 6;17:156. doi: 10.1186/s12936-018-2284-7 (PMC5889563; doi:10.1186/s12936-018-2284-7)
Supplement: Supplementary file 2 — Additional file 2. Geostatistical models formulation. [file 12936_2018_2284_MOESM2_ESM.docx]

**Additional file 2.**

**Geostatistical models formulation**

**Model 1: Estimating malaria risk at cluster level**

Let $y_{i}$ be the number of parasitaemia positive children among the $n_{i}$ screened at cluster$s_{i}, (i=1,\ldots,N)$. We adopt the Bayesian framework of inference and assume that $Y_{i}$ has arisen from binomial distribution, i.e. $Y_{i}|n_{i}, p_{i} \sim Bin(n_{i}, p_{i})$, where the malaria parasite risk at cluster i$p_{i}$, on the logit scale is a function of climatic factors $X_{i}=\left( 1,X_{i1},X_{i2},\ldots X_{ip} \right)^{T}$ , and spatial random effects$U_{i}$, that is$\mathrm{logit} \left( p_{i} \right)= \sum_{k=0}^{p} \beta_{k}X_{ik}+U_{i}$. We assume that $U_{i}$ arise from a spatial process i.e. i.e. $U\sim MVN(\mathbf{0},\Omega)$, where ${{U=(U}_{1},U_{2},\ldots,U_{n})}^{T}$ and $\Omega$is the variance-covariance matrix *NxN*, so that $\Omega_{ij}= \sigma^{2} exp(-\rho d_{ij})$. $d_{ij}$ is the euclidian distance between cluster $s_{i}$ and$s_{j}$, $\sigma^{2}$ is the spatial variance also called the partial sill and $\rho$the smoothing parameter to control correlation. We consider non-informative prior for $\beta_{j}$ defined such as $\beta_{j}\sim$*N*(0,100).

**Model 2: Estimating malaria risk at individual level**

Let $Y_{ij}$ be the status of malaria parasitaemia of child j who lives in cluster$s_{i},(i=1..N)$. We assume a Bernoulli distribution, i.e. $Y_{ij}|p_{ij} \sim Be( p_{ij})$ , and model individual level function covariates on the logit scale of child specific risk,$p_{ij}$, i.e.$\mathrm{logit} \left( p_{ij} \right)= \sum_{k=0}^{p} \beta_{k}X_{ijk}+U_{j}$ where,$X_{ij\boldsymbol{.}}=\left( 1,X_{ij1},X_{ij2},\ldots X_{ijp} \right)^{T}$,the list of covariates including climatic factors and intervention coverage indicators, $U_{i}$ is the dependence spatial effect of cluster $s_{i}$. We use the similar prior distributions for spatial and regression coefficients parameters as above in Model 1.

**Prior distributions of the spatial process parameters**

We assigned to $\sigma^{2}$ an Inverse Gamma prior distribution,$\sigma^{2}\sim IG(2.01,1.01$) and adopt a Uniform prior distribution for 𝜌, i.e. $\rho\boldsymbol{\sim}Unif(\frac{-log(0.05)}{d_{\max}},\frac{-log(0.05)}{d_{\min}}).$The later prior assumes that the spatial correlation bellow 0.05 is negligible, d_max_ and d_min_ were associated to the maximum and minimum (non-zero) euclidean distance between the survey locations.

**Bayesian variable selection**

We applied stochastic search variable selection assuming a normal mixture prior distribution for the regression coefficient of each predictor. A categorical indicator $I_{k}$ is used to exclude the associated predictor from the model when$I_{k}=0$, to select its linear form when $I_{k}=1$ or to select the categorical form when $I_{k}=2$ . We assume a multinomial distribution prior for $I_{k}$ with probability function $\prod_{l=0}^{2} {\pi_{l}}^{\delta_{l}\left( I_{k} \right)}$ , where $\pi_{l}$ represent the inclusion probability of each form (*l* = 0, 1, 2) and $\delta_{k}(.)$ is the Dirac function : $\delta_{k}\left( I_{i} \right)=\left\{ \begin{aligned} 1 if I_{i}=k \\ 0 if I_{i}\neq k \end{aligned} \right.$. We adopt a spike and slab prior for the coefficients $\beta_{k,l}$ that is a mixture of normal prior distribution i.e. $\beta_{k,l}\sim\delta_{l}\left( I_{k} \right)N\left( 0,\tau_{k,l}^{2} \right)+(1-\delta_{l}\left( I_{k} \right))N\left( 0,{c\tau}_{k,l}^{2} \right)$. A non-informative prior Dirichlet distribution with hyper-parameter$\alpha={(1,1,1)}^{T}$is used, then$\pi={(\pi_{1},\pi_{2}, \pi_{3})}^{T}\sim Dirichlet(3,\alpha)$, the constant c is fixed at 10^-5^ and inverse Gamma prior distribution are considered for $\tau_{k,l}^{2}\sim IG(0.01,0.01)$.

**Bayesian kriging**

Let $S_{0}=\{S_{01},S_{02}\ldots,S_{0k}\}$ be the centroids of the gridded surface. We predict the parasitaemia risk at the grid from the predictive posterior distribution (Sudipto Banerjee, 2015):

$$P\left( \tilde{Y_{0}} | \tilde{Y} \right)= \int P\left( \tilde{Y_{0}} | \tilde{Y},\tilde{\beta},\tilde{U_{0}},\tilde{U} \right)P(\tilde{U_{0}}|\tilde{U},\sigma^{2},\rho)P(\tilde{U},\sigma^{2},\rho|Y)P(\tilde{\beta}|\tilde{U},\rho,Y)P(\rho,\tilde{U}|Y)d\beta d\rho dUd\sigma^{2}$$

$\tilde{Y_{0}}=Y\left( S_{0} \right)$ ,where $Y\left( s_{o} \right)\sim Be(N, p_{s_{o}})$ is the predicted number of children tested positive in the new location $S_{o}$ and the associated covariates $X_{0}.$Conditional to the spatial process and model parameters, with the link relation between the risk and spatial covariate defined as $\mathrm{logit} \left( p_{s_{o}} \right)= \sum_{k=0}^{p} \beta_{k}X_{s_{o}k}+U_{0}.$

**References**

1. Ishwaran, H. & Rao, J. S. Spike and slab variable selection: Frequentist and Bayesian strategies. Ann. Stat. 33, 730–773 (2005).
2. O’Hara, R. B. & Sillanpää, M. J. A review of Bayesian variable selection methods: what, how and which. Bayesian Anal. 4, 85–117 (2009).
3. Sudipto Banerjee, Bradley P. Carlin, Alan E. Gelfand, Hierarchical Modeling and analysis for spatial data, Monographs on Statistics and Applied Probability 135, CRC Press, 2015.
